# Feasibility of an incentive scheme to promote active travel to school: a pilot cluster randomised trial

**DOI:** 10.1186/s40814-017-0197-9

**Published:** 2017-11-14

**Authors:** Samuel Ginja, Bronia Arnott, Vera Araujo-Soares, Anil Namdeo, Elaine McColl

**Affiliations:** 1 Institute of Health & Society, Newcastle University, Baddiley-Clark Building, Richardson Road, Newcastle upon Tyne, Tyne and Wear NE2 4AX UK; 2 School of Engineering, Newcastle University, Newcastle upon Tyne, NE1 7RU UK

**Keywords:** Active travel, Schools, Children, Accelerometers, Walking, Cycling, Physical activity, Incentives

## Abstract

**Background:**

In Great Britain, 19% of trips to primary school within 1 mile, and 62% within 1–2 miles, are by car. Active travel to school (ATS) offers a potential source of moderate-to-vigorous physical activity (MVPA). This study tested the feasibility of an intervention to promote ATS in 9–10 year olds and associated trial procedures.

**Methods:**

A parallel cluster randomised pilot trial was conducted over 9 weeks in two schools from a low-income area in northeast England. Measures included daily parental ATS reports (optionally by SMS) and child ATS reports, as well as accelerometry (ActiGraph GT3X+). At baseline, all children were asked to wear the accelerometer for the same week; in the post-randomisation phase, small subsamples were monitored each week. In the 2 weeks when a child wore the accelerometer, parents also reported the start and finish times of the journey to school. The intervention consisted of a lottery-based incentive scheme; every ATS day reported by the parent, whether by paper or SMS, corresponded to one ticket entered into a weekly £5 voucher draw. Before each draw session, the researcher prepared the tickets and placed them into an opaque bag, from which one was randomly picked by the teacher at the draw session.

**Results:**

Four schools replied positively (3.3%, *N* = 123) and 29 participants were recruited in the two schools selected (33.0%, *N* = 88). Participant retention was 93.1%. Most materials were returned on time: accelerometers (81.9%), parental reports (82.1%) and child reports (97.9%). Draw sessions lasted on average 15.9 min (IQR 10–20) and overall session attendance was 94.5%. Parent-child report agreement regarding ATS was moderate (*k* = 0.53, CI 95% 0.45; 0.60). Differences in minutes of accelerometer-assessed MVPA between parent-reported ATS and non-ATS trips were assessed during two timeframes: during the journey to school based on the times reported by the parent (*U* = 390.5, *p* < 0.05, 2.46 (*n* = 99) vs 0.76 (*n* = 13)) and in the hour before classes (*U* = 665.5, *p* < 0.05, 4.99 (*n* = 104) vs 2.55 (*n* = 19)). Differences in MVPA minutes between child-reported ATS and non-ATS trips were also significant for each of the timeframes considered (*U* = 596.5, *p* < 0.05, 2.40 (*n* = 128) vs 0.81 (*n* = 15) and *U* = 955.0, *p* < 0.05, 4.99 (*n* = 146) vs 2.59 (*n* = 20), respectively).

**Conclusions:**

Data suggest the feasibility of an ATS incentive scheme and of most trial procedures. School recruitment stood out as requiring further piloting.

**Trial registration:**

ClinicalTrials.gov: NCT02282631. Registered 5th September 2014.

**Electronic supplementary material:**

The online version of this article (10.1186/s40814-017-0197-9) contains supplementary material, which is available to authorized users.

## Background

Children should engage in at least 60 min of moderate-to-vigorous physical activity (MVPA) per day to improve their physical and psychological health [1]. Yet, most English boys (79%) and girls (84%) aged 5–15 fail to achieve MVPA guidelines [2]. Active travel to school (ATS) may help tackle this problem. High quality studies have suggested that primary school children and high school children accumulate on average, respectively, 17 and 13 min of additional daily MVPA from walking to and from school [3]. Despite these benefits, in Great Britain, 19% of trips to primary school within 1 mile, and 62% within 1–2 miles, are made by car [4]. An existing systematic review has pointed out the need for higher quality study designs and measures before the effectiveness of the ATS interventions can be determined [5]. Besides, many ATS programmes require considerable resources or time from schools, e.g. [6], with clear implications for wider implementation.

An example of relatively inexpensive and easy-to-deliver interventions are incentive schemes. Some data suggest that incentives (e.g. gift vouchers) can effectively promote health behaviours in youth [7, 8], but it is unclear how much this applies to ATS. In a before-after study (US), a lottery-based incentive scheme was successfully implemented and reported to increase cycling to school by 16%, with only 6 cents (approx. £0.05) being spent per child [9]. Methodologically robust studies need to demonstrate the cost-effectiveness of this and similar schemes, taking into account implementation issues in different social and economic contexts.

Following existing guidance on the development and evaluation of complex interventions [10], the present pilot study—RIGHT TRACKS—tested the feasibility of a lottery-based incentive scheme to promote ATS in Year 5 children (age 9–10) and of associated trial procedures. More specific objectives of our study included estimating recruitment, retention and adherence rates for schools and children and informing the selection of an appropriate outcome measure for a future definitive trial. The intervention was underpinned by the Behavioural Ecological Model [11], particularly the idea that behaviour can be changed by introducing more immediate consequences following actions (e.g. receipt of incentives). This model also pays attention to antecedent factors in the social and infrastructural environment of the child, which can be targeted in conjunction with consequence-based strategies.

## Methods

### Study design

The RIGHT TRACKS pilot trial used a cluster randomised design; one school was randomly allocated to the intervention and one school to the control group (no intervention). For this purpose, one member of the research team who was blinded to the specifics of each school tossed a single coin once only. Randomisation only took place at the school level, not at the level of individual participants. See Additional file 1: (CONSORT checklist) for details about the various aspects of this study.

### Study timeline

Schools were recruited in May and June 2014 and children and parents in early September 2014. This timeline allowed the study to begin in the new school term and end before the next school holiday. Pilot trial data were collected from late September to early December 2014 (1 week at baseline and 8 weeks at post-baseline). Ethical approval was granted by the local university research committee and the trial was prospectively registered on clinicaltrials.gov (NCT02282631).

### Measures

Feasibility of the intervention and trial procedures was assessed through a range of indicators such as number of invitations to take part issued (both to schools and to families) and returned; data collection materials issued to participants, returned and completed (including level of completion); logistics of the study sessions and any adjustments required to the study protocol.

A parental baseline questionnaire was adapted from a previous measure used in a UK study in a similar context and age group [12], which assessed individual and family variables, to characterise the study sample and help understand feasibility outcomes (see Additional file 2— Parental Baseline Questionnaire). It was also administrated with the intent of testing the clarity of questions in preparation for an effectiveness trial, in which knowledge of participant characteristics, which might act as confounding factors, mediators or moderators of ATS, would be needed to interpret outcomes (and potentially to inform stratification). No translated versions of study documents were provided considering the very low rates of non-English families in study schools, and consistent with advice from a local head teacher according to whom non-native parents would naturally seek assistance from other families if needed, although it is unknown if this happened in practice in our study.

Three methods were used to assess ATS: parental reports, child reports and accelerometry. Interrater agreement between parental and child reports was assessed, as well as the validity of parental and child report compared to accelerometry on the premise that trips reported as being active (ATS trips) would show higher MVPA than trips reported as non-active (non-ATS trips). If shown to be valid, the sole use of ATS reports could be an option for an evaluation trial considering the high costs of accelerometers, or even the preference for child reports over those by their parents who may be less available to participate in research. The paper parental ATS report form was collected weekly during the pilot trial and had five questions, each with the same format, one for each school day of the week: “On day X, did your child walk or cycle to school, all or part of the journey?”; parents were requested to tick a box (YES or NO) for each question, each day as the week went by. Once completed, the parental ATS report was returned to the classroom by the child on the designated day when the researcher visited the school (same day each week). Optionally, parents could report ATS in real time by SMS, in which case they received a text message each morning with the same question as above and were asked to reply ‘YES’ or ‘NO’.

The questions and format of the child ATS report were similar to that of the parental report. It was collected once a week during the pilot trial and included five yes/no type questions, one for each of the previous five schooldays. The child report was always on paper and completed in the classroom in the researcher’s presence, so that they could be collected immediately without risk of loss; it is acknowledged that this could potentially have resulted in some recall bias.

Children were asked to wear an accelerometer belt (ActiGraph GT3X+) for 7 days (five school days) twice throughout the 9 weeks of the trial, once at baseline and once post-baseline (i.e. intervention phase). At baseline, all participants wore an accelerometer belt simultaneously; whereas in post-baseline weeks, different subsamples (typically two pupils per week) were monitored. This schedule eased data collection for the researcher. Exceptionally during the 2 weeks when the child was fitted with the accelerometer, the parental ATS report was on paper for all participants. This is because, in those 2 weeks, the parent ATS report included additional questions about the start and finish times of each of the five journeys to school and about any pauses during each journey, as well as the usual yes/no questions.

Consistent with previous research (e.g. [13]), the accelerometer was set to collect data at 30 Hz. Data were stored every 10 s (i.e. epoch) [14] for a better capture of intermittent bursts of activity characteristic of young people. The MVPA cut points applied in this study have been demonstrated to be sensitive and specific cut points for sedentary (≤ 100 cpm^1^), light (˃ 100 cpm), moderate (≥ 2296 cpm) and vigorous (≥ 4012 cpm) intensity in children [15].

### School recruitment

Schools approached were primary, junior or middle schools located in the North East of England who were working or had in the past worked with Sustrans, a charity actively involved in ATS promotion in schools [16], as contact details were provided by one of their officers. A first email invitation was sent out to 123 schools, with templates of the study materials. Due to the low number of responses (*n* = 7), the same email invitation was reissued 2 weeks later. After 1 week, schools with pending response (*n* = 108) were contacted by phone. Out of the schools who replied positively, two were selected, taking into account matched characteristics and likely scope for change in ATS based on data about ATS rates at that time provided by Sustrans or the school. This number was deemed sufficient for a pilot study and considering the limited resources available. A meeting was held in each of the participating schools to make arrangements for the start of the study.

### Child and parent recruitment

Children attended a presentation session at school in September 2014 and received a study pack for their parents. Each pack contained a consent form, a parental baseline questionnaire, information leaflets for the parent and child, an information booklet (detailed information about the study) and a prepaid envelope. Parents were requested to complete the consent form and post it to the researcher or leave it at school within 2 weeks. Although parents were also asked to complete the questionnaire, only the completion of the consent form was mandatory for their child’s participation. Children with parental consent completed an assent form in the classroom.

### Baseline and post-baseline assessments

At baseline, each child received a plastic wallet with a blank parental ATS report form, an accelerometer belt and sheet with instructions for use and was instructed to wear the belt around their waist, every day during waking hours for the following 7 days. The paper-based parental ATS report form was taken home by the child, completed by the parent and returned to the classroom by the child along with the accelerometer 1 week later. On returning these materials, children completed their own ATS report for the same week in the classroom.

Measurement procedures for participants wearing the accelerometer each post-baseline week were similar to those followed at baseline. In non-accelerometer weeks, a child was expected to complete the child ATS reports in the classroom and to return the parental ATS report every week if the parent was a paper-respondent. Children whose parent was a SMS respondent only returned a paper-based parental ATS report in the two accelerometer weeks; in the remaining non-accelerometer weeks, those children did not return parental ATS reports. At each accelerometer assessment (i.e. once at baseline and once at post-baseline), control and intervention participants who returned the accelerometer undamaged (irrespective of wear time) and the completed parental and child ATS reports were thanked with a £5 voucher. Other than the incentive scheme in the intervention school, data collection sessions were very similar in both schools.

### Intervention

Underlying our intervention was the assumption that ATS behaviour can be increased more effectively if more immediate consequences follow it, such as material incentives, even if intermittently [17]. The intervention tested in this study consisted of children being entered into a weekly prize draw for walking or cycling to school, all or part of the journey, and was the same for all participants. The prize was one £5 gift voucher (Love2Shop) which could be spent in a number of high street shops. This value approximated the amount of weekly pocket money for children aged 8–15 in the North East of England at the time, £6.23 [18]. It was selected to be sufficiently high to be attractive to the children without being unduly coercive.

Each trip to school reported by the parent as being active corresponded to one ticket with the child’s ID number being placed into the draw which always took place at school. In total, each child could accrue between zero and five tickets per week, depending on the number of active trips to school reported by the parent (i.e. on the five school days) either on paper or by SMS. Unreported or misreported trips could not be carried over from 1 week to the other. Children travelling to school by active modes other than walking or cycling (e.g. scooter, skateboard) were instructed to class themselves as ‘cycling’ to school. Moments before the draws, the researcher (PhD student with an undergraduate degree in Psychology and MSc in Clinical Psychology) produced paper tickets based on the parental ATS reports that children had returned to the school office that morning or based on reports by SMS which were sent directly by the parent to the researcher’s email account. The researcher would then meet participants in a room, separate to their usual classroom and away from non-participants, in the presence of one of the Year 5 teachers (generally the same every week). Children who had not returned their parental ATS form were allowed entry into the draw according to their own ATS report but were reminded that the parental report was necessary for future draws. All the tickets were folded and placed into an opaque bag from which one was picked by the classroom teacher attending the session.

Most of the time in the draw sessions was spent collecting materials, distributing materials and completing the child ATS report. The draws themselves were very brief, 1 or 2 min. The total number of weekly ATS trips in the classroom was represented in a bar chart posted on the wall outside the classroom, to which bars were added week by week. See Additional file 3 for TIDieR checklist.

All eight draw sessions were delivered as intended, but one had to be re-scheduled to a different day on one occasion due to other classroom commitments. Further to a protocol amendment, from the start of the intervention period, children who failed to provide the completed parental ATS report on a draw day were able to take part based on their own report. This protocol amendment happened during the baseline week, when some children failed to return parental forms on time (*n*− = 4). As this was expected to be a recurrent issue in subsequent weeks, the amendment was submitted straight away and approved within a few days.

### Data analysis

Data were analysed in SPSS (IBM Statistical Package for the Social Sciences, version 21.0) and accelerometer data in ActiLife (version 6.11.5). Descriptive statistics, including numbers and percentages, medians and interquartile ranges, were calculated to quantify outcomes of recruitment, retention and adherence to study procedures. Kappa scores (CI 95%) and crude rates of agreement were used to assess parent-child ATS report agreement. Level of agreement based on Kappa scores followed a commonly accepted threshold-based classification according to which agreement can be poor (*k* < 0.00), slight (*k* = 0.00–0.20), fair (*k* = 0.21–0.40), moderate (*k* = 0.41–0.60), substantial (*k* = 0.61–0.80) or almost perfect (*k* = 0.81–1.00) [19]. Mann-Whitney tests were undertaken to compare MVPA between ATS and non-ATS trips (*p* value at 0.05). A scatter plot and Spearman rho were used to assess the association between the duration of ATS trips and MVPA.

The study was not powered for analysis of effectiveness. On request of the journal editor, however, a post hoc analysis was conducted in Stata [20] to estimate the effect size of the intervention. This was attempted with four different outcomes: (a) the minutes of MVPA in the hour before the class in the post-baseline week in which the accelerometer was worn (which varied from pupil to pupil), (b) percentage of MVPA in the hour before the class in the same post-baseline week as above, (c) the number of days on which ATS was reported by the parent during the 40 post-baseline weeks (i.e. 8 weeks) and (d) the number of days on which ATS was reported by the child during the 40 post-baseline days (i.e. 8 weeks). Data on the minutes and percentage of MVPA during the hour before classes were log transformed to meet the condition of data normality. Once log transformed, the results were back transformed to ease interpretation, by calculating *e* to the power of the coefficient. A linear regression model was developed to assess the difference in outcomes (a) and (b) between schools, adjusted for baseline levels of these variables. Assumptions of the linear model were assessed via standardised residual and leverage plots.

Number of ATS days was preferred over percentage of ATS days as the latter was highly skewed. Poisson regression models were used to estimate the difference in outcomes (c) and (d) between schools, adjusted for baseline scores. Assumptions of the Poisson models were assessed via the deviance and Pearson statistic.

## Results

### School recruitment

Schools approached (*n* = 123), mostly community schools (65.9%), had on average (median) 240 students (LQ 206–UQ 362), 3.8% of whom had a first language other than English (LQ 1.9%–UQ 9.2%) and 19.7% of pupils eligible for free meals (LQ 12.1%–UQ 35.6%). Schools took approximately 21 days to reply (LQ 20–UQ 22), including those who replied proactively by email (12.2%) or reactively when contacted by phone (83.7%).

Four schools agreed to take part in the study (3.3%). Compared to all the other schools, these four had (median, IQR) a larger number of students (317, 263–378) and higher proportion of pupils eligible for free meals (31.4%, 10.6–35.7%) but comparable percentage of pupils with a first language other than English (5.0%, 4.9–7.4%). Out of these four schools, two were selected to take part and each had two Year 5 classrooms.

### Individual recruitment and retention

**Fig. 1:**
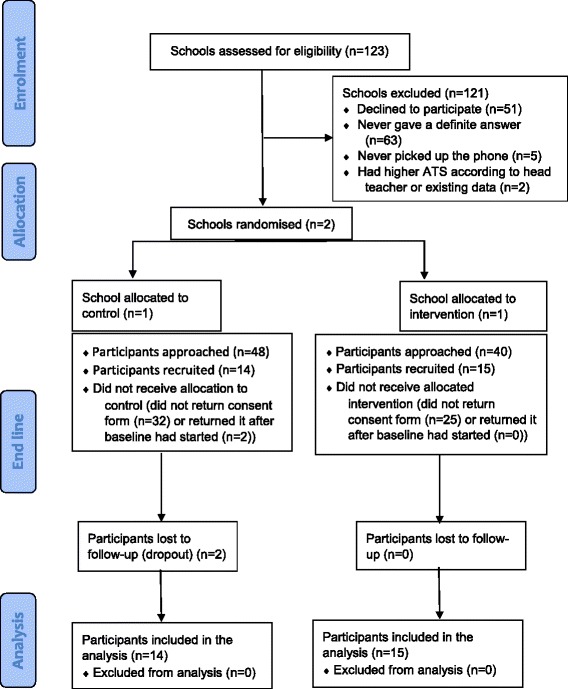
CONSORT flow diagram for the RIGHT TRACKS pilot cluster randomised controlled trial

Parental consent was gained from 29 of the 88 children approached (33.0%) (Fig. 1). Nearly all participants remained for the 9 weeks of the trial in the control school (12/14, 85.7%) and all remained in the intervention school (15/15, 100%). Eight parents opted to report ATS by paper (27.6%), 16 by mobile phone (55.2%); five failed to specify preference and were assigned to the default paper form option (17.2%). No harm was reported by any of the participants, parents or school staff as a result of participating in this study.

### Sample characteristics

Participants (46.4% boys) were predominantly White British (92.6%) and 9 years old (Table 1). Typically three to four people lived in the household and children in the intervention group were more likely to live with two parents/carers than those in the control group (66.7 vs 46.2%). Most children had a car available to drive them to school (75.0%). Parents in the intervention group were more likely to hold higher qualifications than those in the control group, degree/higher degree (40.0 vs 15.4%) or A levels/equivalent (66.7 vs 23.1%). Most parents were employed (82.1%) and the average distance from home to school was half a mile. More children in the intervention school, compared to the control school, were reported to travel to school by car (46.7 vs 25.0%) or to walk (80.0 vs 66.7%) (multiple responses allowed). Most children (75.0%) travelled to school with a parent/carer. Availability of a car to drive the child to school was more likely in the intervention school (80.0%), compared to the control school (69.2%). Both schools’ postcodes were in the fourth quintile in the index of multiple deprivation (IMD), with higher quintiles reflecting higher deprivation, suggesting that participants were mainly from low-income families (data not collected from the families and not presented in Table 1).

**Table 1:** Characteristics of participants in the RIGHT TRACKS study

	Control school	Intervention school	Overall
Gender of the child			
Boy	5 (38.5%)	8 (53.3%)	13 (46.4%)
Girl	8 (61.5%)	7 (46.7%)	15 (53.5%)
Age of the child*	9 (9–9)	9 (9–9)	9 (9–9)
Total number of people living in household*	3 (2–4)	4 (3–4)	3.5 (3–4)
Families with two parents/carers in the household	6 (46.2%)	10 (66.7%)	16 (57.1%)
Car available to drive child to school	9 (69.2%)	12 (80.0%)	21 (75.0%)
Parent’s qualifications**			
Degree or higher degree	2 (15.4%)	6 (40.0%)	8 (28.6%)
A Levels, professional qualification or equivalent	3 (23.1%)	10 (66.7%)	13 (46.4%)
GCSE’s, CSE’s, O Levels or equivalent	11 (84.6%)	13 (86.7%)	24 (85.7%)
None of the above	0 (0.0%)	2 (13.3%)	2 (7.1%)
Ethnic group of parent/carer who completed questionnaire			
White British	11 (91.7%)	14 (93.3%)	25 (92.6%)
Black African	1 (8.3%)	0 (0.0%)	1 (3.6%)
Chinese	0 (0.0%)	1 (6.7%)	1 (3.6%)
Parent who completed questionnaire is employed			
Yes	10 (76.9%)	13 (86.7%)	23 (82.1%)
No	3 (23.1%)	2 (13.3%)	5 (17.9%)
Distance from school (miles) based on postcode*	0.5 (0.3–0.8)	0.5 (0.3–0.7)	0.5 (0.3–0.7)
< 1 mile	9 (83.3%)	13 (86.7%)	23 (85.2%)
1–2 miles	2 (16.7%)	2 (13.3%)	4 (14.8%)
> 2 miles	0 (0%)	0 (0%)	0 (0%)
Child’s travel mode to school on a typical day**			
By car	3 (25.0%)	7 (46.7%)	10 (37.0%)
By bicycle	3 (25.0%)	2 (13.3%)	5 (18.5%)
By walking	8 (66.7%)	12 (80.0%)	20 (74.1%)
Other (scooter)	2 (16.7%)	0 (0.0%)	2 (7.4%)
Who does your child go to school with on most days**			
Child goes alone	1 (7.7%)	3 (20.0%)	4 (14.3%)
With me or my partner	11 (84.6%)	10 (66.7%)	21 (75.0%)
With an older sibling	2 (15.4%)	0 (0.0%)	2 (7.1%)
With other children	3 (23.1%)	2 (13.3%)	5 (17.9%)
With other adults	2 (15.4%)	5 (33.3%)	7 (25.0%)

*Median (IQR)

**Multiple responses allowed

### Adherence to study procedures

Weekly sessions in each school (*n* = 11) included presentation at baseline (*n* = 1), week after baseline (collection of materials before school randomisation) (*n* = 1), post-baseline (including draw sessions in the intervention school) (*n* = 8) and final session (*n* = 1). Only one post-baseline session, in the intervention group, had to be re-arranged to a different day due to school commitments. Initial presentation sessions happened during school hours (i.e. not during break times); thereafter, all sessions at and following baseline were during morning play time in the control school (only assessment in this case) and during school hours in the intervention school (assessment and draw).

After the initial presentation session, only one session in the control school was attended by a staff member (head teacher). In the intervention school, a Year 5 teacher (female) attended all the sessions and actively assisted with study procedures. After the presentation (20 min), the median (IQR) session duration in the control school was 12.5 min (7–15) and in the intervention school 15.9 min (10–20). Overall session attendance was very high, always around or above 90% in both schools.

All accelerometers were returned undamaged, and the return rate of the other ATS measurement materials was above 70% in both groups except the return of parental ATS report forms in the control school (Table 2). Valid reports are those returned with at least 1 day being reported out of the five (i.e. blank forms were excluded from this count). There were eight blank parental ATS forms returned at baseline (four in each school) but no blank forms were ever returned by children or by parents after the baseline week.

**Table 2:** Return of ATS measurement materials in weeks of accelerometer wear and overall

	Control school	Intervention school	Overall
Valid parental ATS reports returned on time in weeks of accelerometer wear	11/26 (42.3%)	26/30 (86.7%)	37/56 (66.1%)
Child ATS reports returned on time in weeks of accelerometer wear	26/26 (100%)	30/30 (100%)	56/56 (100%)
Valid parent ATS report forms available (at all) in the whole study*	32/60 (53.3%)	75/86 (87.2%)	107/146 (73.3%)
Valid child ATS report forms available (at all) in the whole study	108/112 (96.4%)	134/135 (99.2%)	242/247 (97.8%)
*SMS-respondents*: Parent replied to one SMS or more per week	51/53 (96.2%)	56/56 (100%)	107/109 (98.2%)
Accelerometers lost or damaged	0/26 (0%)	0/30 (0%)	0/56 (0%)
Accelerometers returned on time in weeks of wear	19/26 (73.1%)	27/30 (90.0%)	46/56 (82.1%)

*Includes only reports which were returned completed (with at least ATS report on 1 day)

Denominators refer to the number of maximum times on which materials could have been returned (at all) or returned on time

Missing responses on ATS report forms were treated as implied ‘NO’ when only YES’s on that week’s form had been answered, and vice-versa. The same rule applied to parental SMS reports. When there was a mix of YES and NO’s reported within the same week, missing responses were treated as true missing data. The baseline week was the only week where we did not apply the implied response rule, meaning that any blank days on the ATS forms were always treated as true missing data irrespective of what had been reported on the other days that week. This was felt to be an appropriate decision because of an issue with the paper report response format at baseline, which requested participants to circle ‘YES’ or ‘NO’, with ‘YES/NO’ being placed after each of the report questions. This seemed to have contributed to an unusually large number of blank forms, as in the next weeks, the circling format was replaced by a ‘tick the box’ format in which two boxes (one for ‘yes’ and one for ‘no’) were placed below each of the report questions and no more blank forms were returned. On child ATS reports, the response format was also changed after the baseline week in the interests of consistency and clarity although no blank reports had been returned at baseline, probably because the researcher clarified that each question needed responding, when children completed it in the classroom.

In total, across both schools, parental ATS reports provided valid data relating to 979 days (of which 2.8% were implied); there were 261 days with missing data (21.0%), mainly from the control school (183/261). Child ATS reports provided valid data relative to 1150 days (0.2% were implied); there were 90 days (7.3%) with missing data (54/90 from control school).

### Validity of ATS reports

**Fig. 2:**
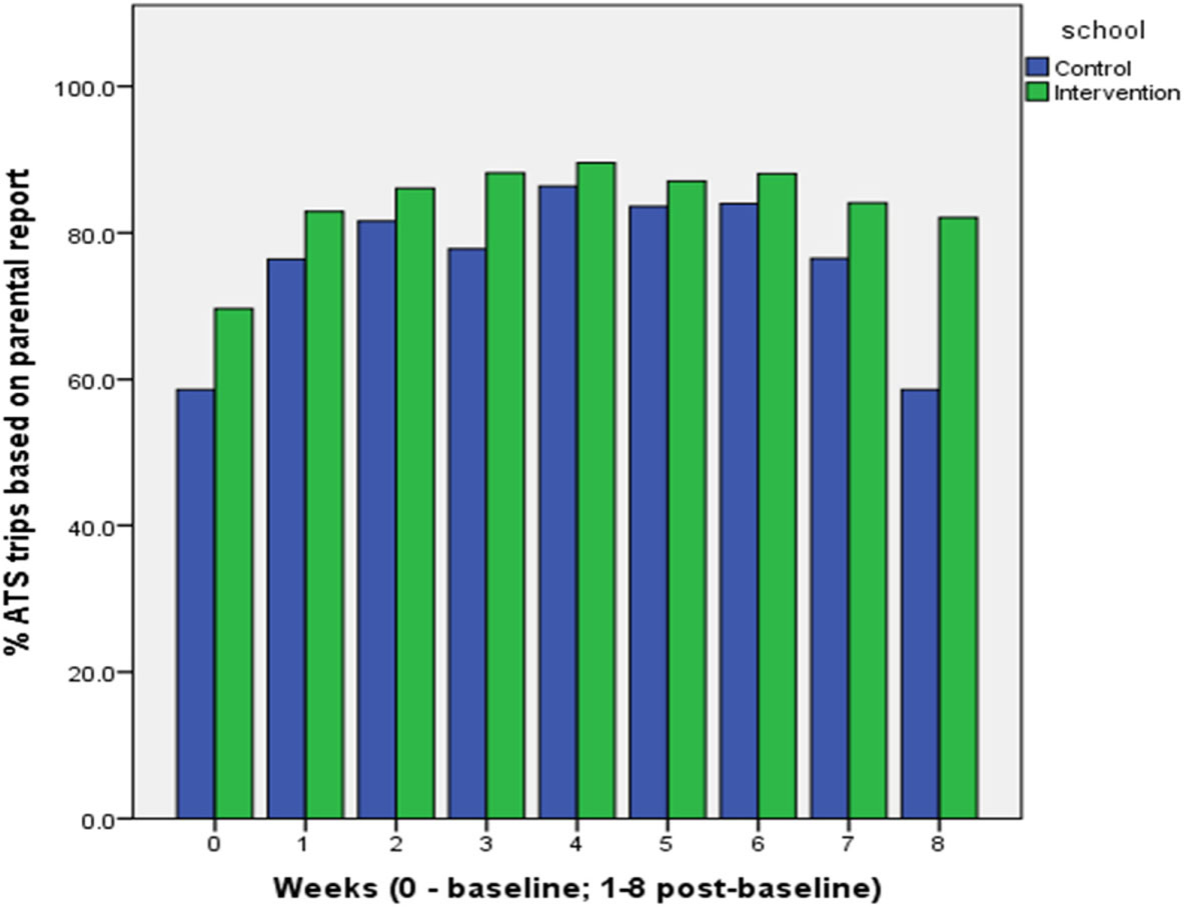
Weekly distribution of ATS trips based on parental report

**Fig. 3:**
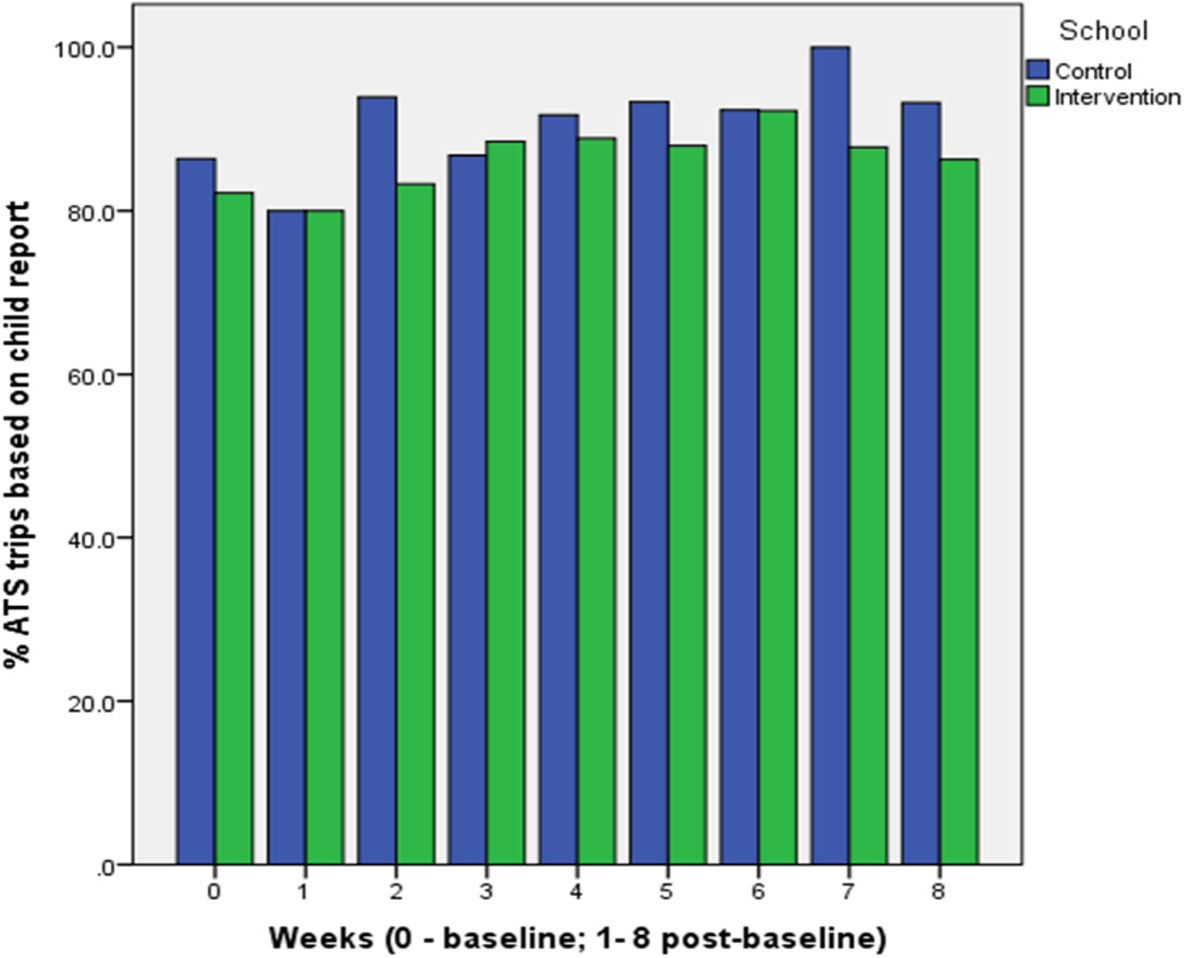
Weekly distribution of ATS trips based on child report

In this study, physical activity was measured objectively through accelerometer-assessed minutes of MVPA. The validity of ATS reports was tested by determining the agreement between parental and child reports, as well as MVPA differences between ATS and non-ATS trips based on parental and child report during two timeframes: the times reported by the parent as pertaining to the times of the journey to school and the hour before classes (7:56 a.m.–8:55 a.m.). The weekly percentage of ATS trips was determined based on parental report (Fig. 2) and based on child report (Fig. 3).

Only cases where both parental and child reports were available were included for interrater agreement analyses. In the control school, there was a fair chance-corrected agreement between parent and child reports, *K* = 0.264, CI 95% 0.138 to 0.384. In the intervention school, there was substantial chance-corrected agreement between both reports, *K* = 0.716, CI 95% 0.635 to 0.791. Overall in both schools, there was moderate chance-corrected agreement between parental and child reports, *K* = 0.526, CI 95% 0.451 to 0.596.

Recordings for MVPA analyses were excluded in the following situations: the accelerometer was not worn at all during the parent-reported times for the journey to school or for the hour before the school starts, accelerometer recordings of less than a 1-min duration (e.g. if the child wore the device for 10 min consecutively and then for 20 s, only the 10-min recording was included), the child was absent from school or parents did not specify times of departure from home and/or arrival at school which made it impossible to examine MVPA during this specific timeframe (although it was still possible to assess MVPA between 7:56 a.m.–8:55 a.m., i.e. the hour before school starts). On average (median, IQR) and equally in both schools, the trip to school lasted 10 min (LQ 6–UQ 15), the duration of accelerometer wear during the times specified by the parents was 10 min (LQ 7–UQ 15) and the percentage of time that the child wore the accelerometer during these times was 100% (LQ 100–UQ 100). However, for this timeframe, there was a considerable number of recordings missing, in the control school (112 missing out of 182, i.e. 61.5%) and in the intervention school (93 missing out of 205, i.e. 45.4%). The median (IQR) value of wear length during the hour before classes was 54 min amongst control participants (24–60), and 60 min amongst intervention participants (51–60), i.e. 90% (40–100) and 100% (85–100) of the designated time, respectively. Again, a large number of recordings were missing for this timeframe, in both the control (85 missing out 182, i.e. 46.7%) and in the intervention school (80 missing out of 205, i.e. 39.0%).

Table 3 shows the MVPA differences between ATS and non-ATS trips. MVPA data were not normally distributed (Shapiro-Wilk *p* < 0.001) but mean (SD) values are presented to ease comparison with other studies in the discussion.

**Table 3:** Differences in MVPA (minutes) between ATS and non-ATS trips

	Based on parent ATS report	Based on child ATS report
MVPA of trips during the times reported by the parent	*U* = 390.5, *p* = 0.02*	*U* = 596.5, *p* = 0.02*
	ATS trips (*n* = 99) 2.46 min (2.83)	ATS trips (*n* = 128) 2.40 min (2.68)
	Non-ATS trips (*n* = 13) 0.76 min (0.95)	Non-ATS trips (*n* = 15) 0.81 min (0.87)
	Missing trips = 170 (60.3%)	Missing trips = 139 (49.3%)
MVPA of trips during the hour before the classes (7:56–8:55)	*U* = 665.5, *p* = 0.02*	*U* = 955.0, *p* = 0.01*
	ATS trips (*n* = 104) 4.99 min (4.11)	ATS trips (*n* = 146) 4.99 min (3.91)
	Non-ATS trips (n = 19) 2.55 min (1.69)	Non-ATS trips (*n* = 20) 2.59 min (1.60)
	Missing trips = 159 (56.4%)	Missing trips = 116 (41.1%)

Based on Mann-Whitney *U* test (Shapiro-Wilk, *p* < .05)

*p < .05

There was a significant difference in the minutes of MVPA between parent-reported ATS trips and non-ATS trips during the times specified by the parent as pertaining to the school journey, *U* = 390.5, *p* < 0.05 (1.7 min difference; 2.46 vs. 0.76 min), and during the hour before the classes, *U* = 665.5 = *p* < 0.05 (2.4 min difference), suggesting that parental reports of ATS trips were indeed valid, since, as expected, those travelling actively exhibited a higher level of MVPA. A significant difference was also found in the minutes of MVPA between child-reported ATS trips and non-ATS trips during the times specified by the parent as pertaining to the school journey, *U* = 596.5, *p* < 0.05 (1.6 min difference), and in the hour before classes, *U* = 955.0, *p* < 0.05 (2.4 min difference), also suggesting the validity of child ATS reports. A larger number of trips with concurrent MVPA data came from the intervention school than from the control school: based on parental report—during the times specified by the parent as corresponding to the school journey 70 vs 42 trips and during the hour pre-classes 78 vs 45; based on child report—during the times specified by the parent 86 vs 57 and during the hour pre-classes, 96 vs 70.

**Fig. 4:**
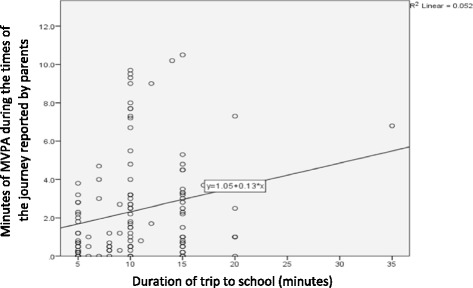
Association between trip duration and MVPA

On the premise that longer trips would result in higher levels of MVPA, a scatter plot was used to depict the relationship between the duration of the trip to school based on parental ATS report and the minutes of MVPA during the parent-reported times of the trip to school (Fig. 4). There was a wide range of MVPA values associated with some commonly reported trip durations, e.g. 10 min long trips. Only 5.2% of the variability in MVPA was explained by trip duration. The Spearman rho value of 0.27 indicates a weak relationship [21]. Together these findings suggest that duration of the journey to school as reported by parents has little impact on MVPA amassed.

### Estimated effect size of intervention

Based on data from 26 children, mean minutes of MVPA during the hour before classes in the post-baseline week decreased by 3.0% on average in the intervention school compared to control school (95% CI − .47 to .41). Similarly, mean percentage of MVPA during the hour before classes in the post-baseline week decreased by 25.0% in the intervention school compared to control (95% CI − .87 to .30). After testing the model assumptions using leverage and residual plots, two outlying values of percentage of MVPA were identified. Excluding those two observations, mean percentage of MVPA during the hour before classes in the post-baseline week was found to decrease by 16.6% in the intervention school, compared to control school (95% CI − .60 to .23).

Out of 27 participants who were retained for the whole duration of the study, 10 of them failed to return a valid parental report at baseline (six in the control group) and as such could not be included in an analysis of parent-reported ATS. Data from these 17 children indicated that the rate of parent-reported ATS days was 1.10 times higher in the intervention school compared to control (incidence rate ratio (IRR) = 1.10; 95% CI 0.90 to 1.34).

Data from 27 children showed that the rate of child-reported ATS days reduced by a factor of 0.99 in the intervention school compared to control (IRR = 0.99; 95% CI 0.86 to 1.13).

## Discussion

This study investigated the feasibility of a lottery-based incentive scheme to promote ATS and associated trial procedures. Once enrolled, all 15 participants in the intervention group were retained for the whole duration of the study; attendance at draw sessions was excellent, as was the provision of parental reports for the draws. Most trial procedures were feasible in both schools, as indicated by the very good rates of return of outcome data collection including parental ATS reports, child ATS reports and accelerometers, as well as by overall retention and session attendance. However, one particular aspect of the trial—the strategy used to recruit schools—stood out as not being feasible, as only 3.3% of schools contacted replied positively. This is much lower than that reported in studies of similar context and age group [22–24]. In those studies, school representatives were approached personally at school or through a head teacher network. Unfortunately, contact details for head teacher groups in the areas in which the research was carried out were unavailable to us. The fact that ours was a student-led project, with no remuneration for schools, could also have made participation less appealing to the schools approached.

The participant recruitment rate (33.0%) was also comparatively low (e.g. [25, 26]), possibly due to the amount of paper work and parental involvement required in our study, factors previously identified as having a deterring effect on pupil recruitment [27]. An opt-out procedure might also have led to better recruitment [28]. It is possible also that those families who lived further from the school and for whom ATS might have been less convenient chose not to participate.

Participant retention and accelerometer return were excellent compared to previous ATS studies (e.g. [29, 30]). A possible reason for this were the £5 thank you vouchers issued to all participants who returned materials when requested, consistent with findings on high retention strategies among low-income families [31]. The small sample size, which allowed the researcher to have more contact with participants and to remind them personally to return the device when necessary, may also have contributed to the perfect rate of return of accelerometers. Other aspects of adherence to procedures were also satisfactory, such as session attendance and provision of ATS reports, although fewer parental reports were available on paper than by SMS. This aligns with findings from a previous study where SMS reports (both by children and parents) showed lower attrition (28%) than paper diaries (61%) and greater adherence to intervention (43 vs 19%, *p* < 0.02) [32]. Availability of MVPA recordings during the two timeframes considered in our study, 64.4 and 78.7%, is difficult to compare with that of previous research because such information is usually unreported (e.g. [33, 34]).

Most feasibility outcomes were more favourable in the intervention school than in the control school, including participant recruitment and retention, return of children’s and parental ATS reports, timely return of accelerometers and provision of MVPA data, session attendance and interrater agreement. Besides chance, this could be attributable to participant baseline differences, as lower adherence to study procedures has been reported amongst children from lone parent families or from less favourable socioeconomic circumstances [35], both of which were more prevalent in the control group compared to intervention group in our study (lower socioeconomic status was suggested by lower car availability and fewer parents with high educational qualifications). Another potential reason was that intervention participants were more enthusiastic about the study because they received the intervention, but this would not explain pre-randomisation differences (e.g. rate of participation and rates of return of data at baseline, which occurred pre-randomisation). The fact that data collection happened during morning play time in the control school, and not during classroom hours as in the intervention school, could also have had a negative impact as control children could have been less eager to spend part of their break in the classroom. Another possible explanation was the involvement of school staff, particularly the Year 5 teacher, which was strong in the intervention school but minimal in the control school.

Significant differences in minutes of MVPA were found between ATS and non-ATS trips during the times specified by the parents and in the hour before the classes, regardless of whether reports were based on parental reports or on children’s. The fact that the interrater agreement was poorer in the control school probably had little effect on MVPA analyses because the majority of data used for MVPA analyses was from participants in the intervention school. It is unknown how the return of parental ATS paper forms by children affected the agreement between the two sources, if at all, but the occurrence of tampering seems unlikely for at least two reasons: parental ATS reports by SMS, received directly from parents’ mobile phones, yielded the same ATS rates as those from paper reports, 81.1% (426 trips) vs 81.5% (374 trips), respectively, and parental paper reports were generally completed in pen and crossings out were rare. Overall, findings suggest that reports from both sources are valid at a similar level.

To the best of our knowledge, this study is the first to assess the validity of ATS reports during a parent-reported timeframe. The MVPA difference found between ATS and non-ATS trips during timeframe (1.6 to 1.7 min) was small considering the average duration (10 min) and distance (0.5 miles) of the commute to school in our study (in other words, a child walking all the way to school would be expected to exhibit considerably higher levels of MVPA in a 10-min walk). This could be due to a misreport of the actual times of the journey which would have resulted in physical activity being assessed at the wrong times, as well as to a misreport of travel mode (i.e. non-ATS trips reported as ATS or vice-versa, or part-ATS trips being reported as non-ATS). Still, when using a wider and fixed timeframe—the hour before school—we found a 2.4-min MVPA difference between ATS and non-ATS trips in our study which is below that usually reported in studies with similar measurement procedures, e.g. 5.6 min [36]. A possible explanation for this discrepancy, besides the misreport of travel mode, is the encouragement of ‘park and stride’ trips in the intervention school, from which most accelerometer data was obtained. These partway active trips were allowed entry into the weekly draws, possibly resulting in large spreads of MVPA values for trips of identical duration (Fig. 4).

Based on the above findings, we recommend using a wider and fixed timeframe to detect MVPA differences between ATS and non-ATS trips, as opposed to reported journey times. Although problems exist with both timeframes, the difference between ATS and non-ATS trips found in the hour before school (2.4 min) is likely to be nearer to what would be expected of a 10-min walk than the MVPA difference observed here in the parent-reported times of the journey (1.6–1.7 min). This is supported by a study conducted in the UK (*n* = 141, age 11–12) which combined a Global Positioning System (GPS) with accelerometry and found that approximately half of the journey was spent on MVPA [37]. More precisely, the average duration of the trip to school in that study was 20.3 min and children who walked to school accumulated on average 10.5 of MVPA during the journey to school, which was also true for the afternoon trip [37]. In the same study, children who walked to school also accumulated on average 14.5 min of MVPA in the hour before classes. By the same token, we would expect to obtain 5 min of MVPA during ATS trips because the average trip duration in our study was 10 min and some extra MVPA time in the hour before the classes, which was not the case. Unlike Sowthward et al., we assessed trips and their duration by report and are unable to distinguish uni- and multi-mode trips.

At the request of the editor of this journal, we conducted a post hoc analysis of the estimated effect size of the intervention on four possible outcomes: minutes of MVPA during the hour before school, percentage of MVPA during the hour before school, number of ATS days reported by the parent out of the 40 post-baseline days and number of ATS days reported by the child out of the 40 post-baseline days. None of the four regression models yielded statistically significant results but, to our surprise, three of them suggested a decrease of the outcome in the intervention. Only one of the models—the one where the outcome was the number of ATS days reported by the parent—suggested an increase in the intervention school, and this was the model with the smallest number of participants included (*n* = 17). It is difficult to interpret these results. We believe that a larger sample size is needed to assess the effects of the intervention on any of the four outcomes and recommend caution in the interpretation of our intervention effect size estimates.

Our study had a number of limitations. The sample size was small and, in keeping with the pilot nature, meant that it was not adequately powered for an analysis of effectiveness. Study participants were predominantly White British, from a relatively deprived area (both schools were in the fourth IMD quintile), making it difficult to generalise findings to other populations. However, we were able to successfully engage with participants in these areas, which is often challenging. Although an objective measure of physical activity, accelerometry does not provide the contextual information necessary for an exclusive focus on ATS behaviour and the resort to reported measures is needed. Due to limited resources, only one researcher (PhD student) was involved in the delivery of procedures and data analysis.

## Conclusion

In conclusion, an ATS incentive scheme with an approximate duration of 3 months seems feasible for those participants who took part. However, a different recruitment strategy, especially at the school level, is necessary and should be further investigated. Most materials were returned by participants, but parental ATS reports by SMS, rather than on paper, may be a better option. Selecting a wider and fixed timeframe for analysis of the journey to school (e.g. hour before the classes) may allow a more accurate detection of MVPA differences between ATS and non-ATS trips. Both parent and child ATS reports appear to be valid against accelerometry, but further validation work must be undertaken if the aim is to identify clearer differences between active and non-active trips to school. The high levels of ATS reported by parents and children suggest that an evaluation trial may benefit from targeting schools and children where the scope for ATS promotion is greater, based on updated and accurate data. Family circumstances, local environment and infrastructure and school policies may act as confounding factors, mediators and moderators, and data need to be collected in a consistent manner on these variables. How family background, receipt of an intervention and school involvement affect feasibility outcomes should be further explored; for this purpose, a ‘realist trial’ perspective [38] may be appropriate.

## Additional files

Additional file 1: CONSORT Checklist. (DOCX 29 kb)

Additional file 2: Parental Baseline Questionnaire. (DOCX 328 kb)

Additional file 3: TIDieR Checklist. (DOCX 12 kb)

## Footnotes

1 Counts per minute (cpm) are a product of the amplitude and frequency of the vertical acceleration of the body, used as an indicator of amount and intensity of physical activity.
