# Supplementary material for: Feasibility of an incentive scheme to promote active travel to school: a pilot cluster randomised trial
Source: Pilot Feasibility Stud. 2017 Nov 14;3:57. doi: 10.1186/s40814-017-0197-9 (PMC5686940; doi:10.1186/s40814-017-0197-9)
Supplement: Supplementary file 2 — Parental Baseline Questionnaire. (DOCX 328 kb) [file 40814_2017_197_MOESM2_ESM.docx]

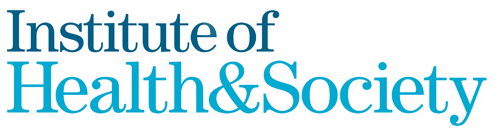

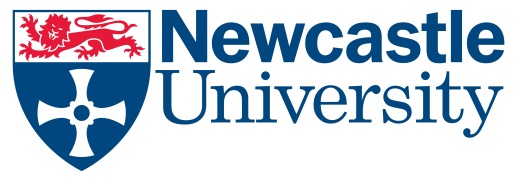


Questionnaire ID

**Parent/Carer Questionnaire**


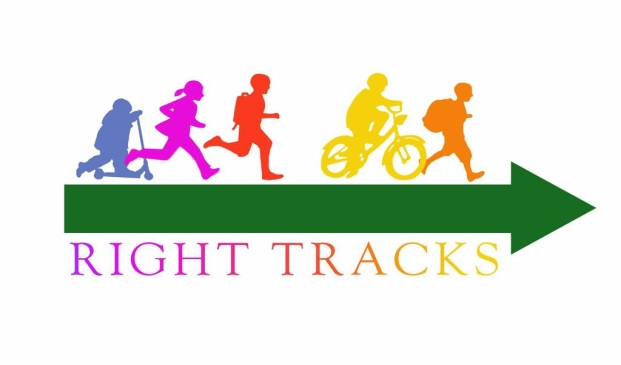


- The questions in this questionnaire relate to you or your Year 5 child.

- Completing this questionnaire will take approximately 30 minutes. If you prefer, you can stop part-way through and come back to it later.

- Please answer the questions as honestly and accurately as you can.

- If you make a mistake, please cross out the incorrect answer and give a new answer.

- Your answers will be treated as confidential.

- If you have any questions, please contact the RIGHT TRACKS study team:

Researcher’s name: Mr Samuel Ginja

Institute of Health & Society, Newcastle University

Baddiley-Clark Building, Richardson Road,

Newcastle upon Tyne, NE2 4AX

**Telephone: 0191 208 6459**

**Section 1: Parental travel and physical activity**

The following set of questions is about the parent(s)/carer(s) who **live with the Year 5** child most time. If applicable, please ask your partner to answer these questions where appropriate or discuss the answers with him/her.

**PARENT/CARER’S ACTIVITIES**

1. This questionnaire is being completed by: *Please tick the box that applies.*
   1. Mother
   2. Father
   3. Other (e.g. grandparent, male carer, please describe): _______________________________________
2. Are you currently employed? *Please tick the box that applies.*
   1. Yes – **Go to question 3**
   2. No – **Go to question 4**
3. How do **you** normally travel to work? *Please tick the option that applies, or options that apply if more than one, and write in the time spent (in minutes) for each travel mode used.*

*Travel mode Time spent on average (min)*

- 1. By car _________
  2. By public transport _________
  3. On foot _________
  4. By bicycle _________
  5. Work from home

1. In a typical week during the past 4 weeks, how many hours did **you** spend on each of the following activities? *Please tick an option for each item.*

0h 0-2h 2-4h ˃4h

- 1. Walking, including walking to work, shopping and leisure
  2. Cycling, including cycling to work and during leisure time
  3. Other physical exercise (such as keeping fit, aerobics,

swimming, jogging).

1. Do you live with a partner? *Please tick the box that applies.*
   1. Yes - **Go to question 6**
   2. No - **Go to question 10**

**OTHER PARENT/CARER’S ACTIVITIES**

1. What is **your partner’s relationship** to the Year 5 child? *Please tick the box that applies.*
   1. Mother
   2. Father
   3. Other (e.g. male carer), please describe: _______________________________________
2. Is your partner currently employed? *Please tick the box that applies.*
   1. Yes – **Please go to question 8**
   2. No – **Please go to question 9**
3. How does **your partner** normally travel to work? *Please tick the option that applies (or options that apply) and write in the time spent (in minutes) for each travel mode used.*

*Travel mode Time spent on average (min)*

- 1. By car _________
  2. By public transport _________
  3. On foot _________
  4. By bicycle _________
  5. Work from home

1. In a typical week during the past 4 weeks, how many hours did **your partner** spend on each of the following activities? *Please tick an option for each item.*

0h 0-2h 2-4h ˃4h

- 1. Walking, including walking to work, shopping and leisure
  2. Cycling, including cycling to work and during leisure time
  3. Other physical exercise (such as keeping fit, aerobics,

swimming, jogging).

**Section 2 – Your Year 5 child’s travel modes**

1. What is the gender of your Year 5 child? *Please tick the box that applies.*
   1. Boy
   2. Girl
2. How old is your Year 5 child? *Please write number in the box*.

Years

1. Does your child have a condition that affects his/her ability to be physically active? *Please tick the box that applies.*
   1. No
   2. Yes. Please describe: _____________________________________________
2. Can your child ride a bike? *Please tick the box that applies.*
   1. Yes
   2. No
3. Does your child have regular access to a bike? *Please tick the box that applies.*
   1. Yes
   2. No
4. How far does your child need to travel to get to school? *Please tick the box that applies.*
   1. Less than 1 mile
   2. 1-2 miles
   3. 2-3 miles
   4. Over 3 miles
5. How does your child travel to school on a typical day?

*Please tick the option that applies, or options that apply, and write in the time spent (in minutes) for each travel mode used.*

*Travel mode Time spent on average (min)*

1. By car ________
2. By school bus ________
3. By public transport ________
4. By bicycle ________
5. By walking ________
6. Other: _________________ ________
7. Who does your Year 5 child go to school with on most days? *Please tick all the options that apply*
   1. My child goes alone.
   2. With me or my partner.
   3. With an older sibling (brother or sister).
   4. With other children (e.g. friends, neighbours).
   5. With other adult(s) (e.g. carer, other parents).
   6. Other. Please describe: ____________________________________
8. How does your child travel from school on a typical day?

*Please tick the option that applies, or options that apply, and write in the time spent (in minutes) for each travel mode used until arrival at home.*

*Travel mode Time spent on average (min)*

1. By car ________
2. By school bus ________
3. By public transport ________
4. By bicycle ________
5. By walking ________
6. Other: _________________ ________
7. Who does your Year 5 child return from school with on most days? *Please tick all the options that apply*
   1. My child returns from school alone.
   2. With me or my partner.
   3. With an older sibling (brother or sister).
   4. With other children (e.g. friends, neighbours).
   5. With other adult(s) (e.g. carer, other parents).
   6. Other. Please describe: ____________________________________
8. Who decides whether your child walks (or cycles) to school or not? *Please tick the box that applies*
   1. Parent(s)/carer(s)
   2. Child
   3. Other. Please describe: _______________________________
9. How often does your Year 5 child walk to the following places? *Please tick one box on each line*

*None*

*within*

*walking Less than 1 to 3 times 4 to 5 times 6 or more*

*Never distance once a week per week per week times per week*

1. Friend’s house
2. Parks or playgrounds
3. Shops
4. Sports venue
5. How often does your Year 5 child ride a bike to the following places? *Please tick one box on each line*

*None*

*within*

*walking Less than 1 to 3 times 4 to 5 times 6 or more*

*Never distance once a week per week per week times per week*

1. Friend’s house
2. Parks or playgrounds
3. Shops
4. Sports venue

**Section 3 – Your perceptions and attitudes towards active travel**

1. Below are a number of statements that might be made about your child’s journey to school. Please circle the number that best indicates your agreement or disagreement with *each statement.*

1 2 3 4 5

Neither

Strongly Disagree disagree Agree Strongly

disagree nor agree agree

- 1.
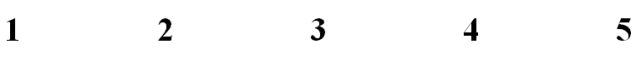
The traffic makes it too dangerous for my child to walk to school.
  2.
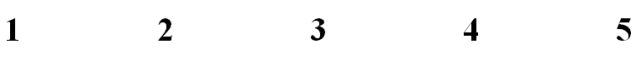
The traffic makes it too dangerous for my child to cycle to school.

1 2 3 4 5

Neither

Strongly Disagree disagree Agree Strongly disagree nor agree agree

- 1.
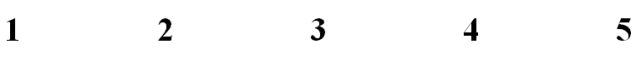
My child cannot walk to school as it’s too far away.


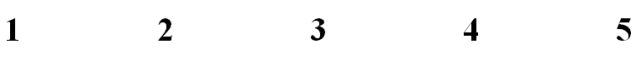


- 1. My child cannot cycle to school as it’s too far away.
  2.
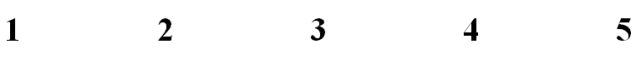
It is more convenient to take my child to school by car.
  3.
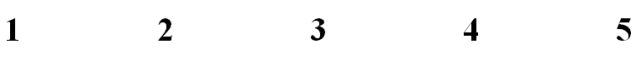
I am worried that something will happen to my child on the way to school.


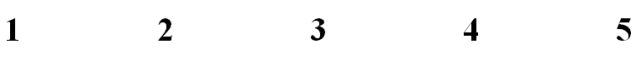


- 1. I’m usually around to take my child to school.


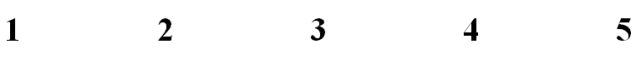


- 1. I take my child to school on the way to somewhere (e.g. work).


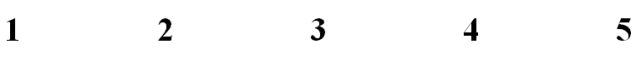


- 1. There are no safe cycle paths on the way to school.
  2.
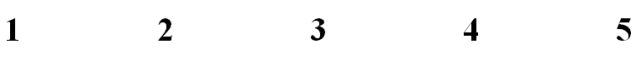
There are no safe pavements on the way to school.


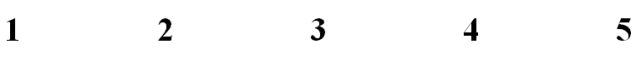


- 1. I like or would like my child to walk to school.


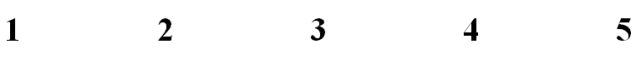


- 1. I like or would like my child to cycle to school.
  2.
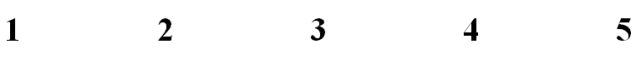
If walking or cycling, my child may walk past convenience stores and buy unhealthy snacks or drinks.
  3.
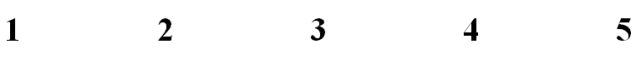
If walking or cycling, my child is more likely to engage in harmful or anti-social behaviours (e.g. smoking, vandalism or bullying others).


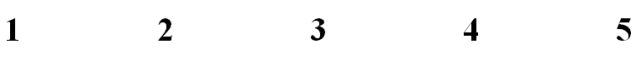


- 1. If walking or cycling, my child is more likely to be bullied by others.

1. Below are a number of statements that might be made about your local area. Where mentioned ‘within easy walking distance’ means within a 10-15 minute walk from your home. *Please circle the number that best indicates your agreement or disagreement with each statement.*

|  | **1**  **Strongly disagree** | **2**  **Somewhat**  **disagree** | **3**  **Somewhat**  **agree** | **4**  **Strongly**  **agree** |
| --- | --- | --- | --- | --- |
| 1. a. There are shops to visit within easy walking distance of my home. | 1 | 2 | 3 | 4 |
| 1. b. There is a park or open space to visit within easy walking distance of my home. | 1 | 2 | 3 | 4 |
| c. There is a sports or leisure centre within easy walking distance of my home. | 1 | 2 | 3 | 4 |
| d. It is pleasant to walk in my local area. | 1 | 2 | 3 | 4 |
| e. There are pedestrian crossings to help walkers cross busy streets in my local area. | 1 | 2 | 3 | 4 |
| f. I feel generally safe walking in my local area. | 1 | 2 | 3 | 4 |
| g. The crime rate in my local area makes it unsafe to go on walks at night. | 1 | 2 | 3 | 4 |
| h. It is easy to walk to a bus stop from my home. | 1 | 2 | 3 | 4 |
| 1. There are few cul-de-sacs (dead-end streets) in my local area. | 1 | 2 | 3 | 4 |
| 1. j. There are a lot of busy junctions in my local area. | 1 | 2 | 3 | 4 |
| 1. k. There are major barriers to walking in my local area that make it hard to get from place to place (for example, busy roads, railway lines, rivers, hills). | 1 | 2 | 3 | 4 |
| 1. l. There are many alternative routes for getting from place to place in my local area. (I don’t have to go the same way every time). | 1 | 2 | 3 | 4 |
|  | **1**  **Strongly disagree** | **2**  **Somewhat disagree** | **3**  **Somewhat agree** | **4**  **Strongly agree** |
| 1. m. There are pavements on most of the streets in my local area. | 1 | 2 | 3 | 4 |
| 1. n. There are cycle paths in or near my local area that are easy to get to. | 1 | 2 | 3 | 4 |
| 1. o. There is a verge that separates the streets from the pavements in my local area. | 1 | 2 | 3 | 4 |
| 1. p. There are trees along the streets in my local area. | 1 | 2 | 3 | 4 |
| 1. q. There are diverse and interesting things to look at in my local area (e.g. buildings and views). | 1 | 2 | 3 | 4 |
| 1. r. There is so much traffic along nearby streets that it makes it difficult or unpleasant to walk in my local area. | 1 | 2 | 3 | 4 |
| 1. s. There is so much traffic along nearby streets that it makes it difficult or unpleasant to cycle in my local area. | 1 | 2 | 3 | 4 |
| 1. t. The speed of traffic on most nearby streets is usually slow (30 mph or less). | 1 | 2 | 3 | 4 |
| 1. u. Most drivers exceed the posted speed limits while driving in my local area. | 1 | 2 | 3 | 4 |
| 1. v. My local area streets are well lit at night. | 1 | 2 | 3 | 4 |
| 1. w. Walkers and cyclists on the streets in my local area can be easily seen by people in their homes. | 1 | 2 | 3 | 4 |
| x. There is a high crime rate in my local area. | 1 | 2 | 3 | 4 |

1. How confident are you that your child can…

*Please circle the number that best indicates your agreement or disagreement with each statement.*

| \|  \| \| --- \| | **1**  **Not at all**  **Confident** | **2**  **Not Particularly Confident** | **3**  **Somewhat Confident** | **4**  **Quite Confident** | **5**  **Very Confident** |
| --- | --- | --- | --- | --- | --- | --- |
| a. Walk to school | 1 | 2 | 3 | 4 | 5 |
| b. Ask a parent or other adult to walk to school with them | 1 | 2 | 3 | 4 | 5 |
| c. Ask a friend to walk to school with them | 1 | 2 | 3 | 4 | 5 |
| d. Walk to school even if their friends don’t walk | 1 | 2 | 3 | 4 | 5 |
| e. Walk to school in bad weather | 1 | 2 | 3 | 4 | 5 |
| f. Cross difficult roads when walking to school | 1 | 2 | 3 | 4 | 5 |
| g. Cope with busy traffic when walking to school | 1 | 2 | 3 | 4 | 5 |
| h. Walk to school even if there are many cars near the school entrance | 1 | 2 | 3 | 4 | 5 |
| i. Walk to school even if there are not enough lollipop people | **1**  **Not at all**  **Confident**  1 | **2**  **Not Particularly Confident**  2 | **3**  **Somewhat Confident**  3 | **4**  **Quite Confident**  4 | **5**  **Very Confident**  5 |
| j. Walk to school even if they are frightened of meeting strangers | 1 | 2 | 3 | 4 | 5 |
|  |  |  |  |  |  |
| k. Walk to school even if they are frightened of being bullied | 1 | 2 | 3 | 4 | 5 |
| l. Walk to school even if there is poor lighting | 1 | 2 | 3 | 4 | 5 |
| m. Walk to school even if it takes a long time | 1 | 2 | 3 | 4 | 5 |
| n. Find a route to walk to school | 1 | 2 | 3 | 4 | 5 |

If your child is unable to ride a bike or does not have a bike, please skip to question 27.

1. How confident are you that your child can…

*Please circle the number that best indicates your agreement or disagreement with each statement.*

| \|  \| \| --- \| | **1**  **Not at all**  **Confident** | **2**  **Not Particularly Confident** | **3**  **Somewhat Confident** | **4**  **Quite Confident** | **5**  **Very Confident** |
| --- | --- | --- | --- | --- | --- | --- |
| a. Cycle to school | 1 | 2 | 3 | 4 | 5 |
| b. Ask a parent or other adult to cycle to school with them | 1 | 2 | 3 | 4 | 5 |
| c. Ask a friend to cycle to school with them | 1 | 2 | 3 | 4 | 5 |
| d. Cycle to school even if their friends don’t cycle | 1 | 2 | 3 | 4 | 5 |
| e. Cycle to school in bad weather | 1 | 2 | 3 | 4 | 5 |
| f. Cross difficult roads when cycling to school | 1 | 2 | 3 | 4 | 5 |
| g. Cope with busy traffic when cycling to school | 1 | 2 | 3 | 4 | 5 |
| h. Cycle to school even if there are many cars near the school entrance | 1 | 2 | 3 | 4 | 5 |
| i. Cycle to school even if there are not enough lollipop people | **1**  **Not at all**  **Confident**  1 | **2**  **Not Particularly Confident**  2 | **3**  **Somewhat Confident**  3 | **4**  **Quite Confident**  4 | **5**  **Very Confident**  5 |
| j. Cycle to school even if they are frightened of meeting strangers | 1 | 2 | 3 | 4 | 5 |
|  |  |  |  |  |  |
| k. Cycle to school even if they are frightened of being bullied | 1 | 2 | 3 | 4 | 5 |
| l. Cycle to school even if there is poor lighting | 1 | 2 | 3 | 4 | 5 |
| m. Cycle to school even if it takes a long time | 1 | 2 | 3 | 4 | 5 |
| n. Find a route to cycle to school | 1 | 2 | 3 | 4 | 5 |

**Section 4: About your family and your household**

*This section will allow us to describe the families in our study. Questions are about the parents or carers of your Year 5 son/daughter now. If applicable, please ask your partner to answer these questions where appropriate or discuss the answers with him or her.*

*Question 27 can help us study important factors of the environment around your child’s home and route to school, such as busy roads, quality of pavement and air quality. We would appreciate if you could provide us with the full postcode of the address, and remind you that all data are confidential and will only be accessed by the research team.*

1. What is the postcode for the home address where your Year 5 child spends most time?
2. How many people live in the household in total? *Please write number in the box*.
3. How many young people in each of the age bands are there in the household (including young person taking part in the study)? *Please write a number in each box*.

|  | 0-3 years |
| --- | --- |
|  | 4-6 years |
|  | 7-11 years |
|  | - 1. years |

1. Is there a car available to drive your child to school? *Please tick only one option.*
   1. Yes
   2. No
2. Is there a car available to drive your child from school? *Please tick only one option.*
   1. Yes
   2. No
3. Do you have any of the following qualifications? *Please tick all that apply*.
   1. Degree or Higher Degree
   2. A Levels, professional qualification or equivalent
   3. GCSE’s, CSE’s, O Levels or equivalent
   4. None of the above
4. What is the ethnic group of the parents/carers of your Year 5 son/daughter? *Please write the appropriate letter in the boxes below*.

*White* *Mixed*

A – British D – White & Black Caribbean

B – Irish E – White & Black African

C – Any other white background F – White & Asian

G – Any other mixed background

*Asian Black or Black British Chinese or other ethnic group*

H – Indian M – Black Caribbean P - Chinese

J – Pakistani N – Black African Q – Other ethnic group

K – Bangladeshi O – Other Black

L – Any other

Asian background

Ethnic group of parent/carer 1:

Ethnic group of parent/carer 2 (*if applicable*):

This is the end of the questionnaire. Please check that you have answered all the questions.

**THANK YOU FOR COMPLETING THIS QUESTIONNAIRE. PLEASE SEAL IN THE ENVELOPE PROVIDED AND RETURN TO THE CLASSROOM ON, OR BY POST BY MONDAY 15^th^ SEPTEMBER**
