# Supplementary material for: Feasibility of an incentive scheme to promote active travel to school: a pilot cluster randomised trial
Source: Pilot Feasibility Stud. 2017 Nov 14;3:57. doi: 10.1186/s40814-017-0197-9 (PMC5686940; doi:10.1186/s40814-017-0197-9)
Supplement: Supplementary file 3 — TIDieR Checklist. (DOCX 12 kb) [file 40814_2017_197_MOESM3_ESM.docx]

Additional file – main aspects of the intervention tested in this study according to the TIDieR checklist (Hoffman et al, 2014)

| 1. Brief name | Lottery-based incentive scheme to promote active travel to school (ATS) |
| --- | --- |
| 2. Why (rationale and theory) | Many of the reinforcing consequences of ATS are long deferred. According to operant conditioning, ATS behaviour can be made more reinforcing if more immediate consequences follow it, such as material incentives. |
| 3. Materials | - £5 gift vouchers (Love2Shop), spendable in a number of high street shops  - Parental ATS report, by paper or by SMS  - Child ATS report |
| 4. Procedures | - Children who walked or cycled to school, all or part of the journey, were entered into a weekly prize draw.  - The prize was a £5 gift voucher.  - Each morning school trip on foot or by bicycle reported by the parent, either on paper or by text message, corresponded to one ticket with the child’s ID on placed into the draw.  - In total, each child could accrue between zero and five tickets per week, depending on the number of active trips to school reported by the parent (i.e. five school days).  - Unreported or misreported trips could not be carried over from one week to the other.  - Children travelling to school by active modes other than walking or cycling (e.g. scooter, skateboard) were instructed to class themselves as ‘cycling’ to school. |
| 5. Who provided | - PhD student; BSc in Psychology, MSc in Clinical Psychology. |
| 6. How | - Delivered face-to-face to the whole group. |
| 7. Where | - In the classroom, at school |
| 8. When and how much | - There were eight draw sessions with an average duration of 14min each, between October and December 2014. Most of the draw sessions were spent collecting materials, distributing materials and completing the child ATS report. The draws themselves were very brief, one or two minutes. |
| 9. Tailoring | - The intervention was not developed to be personalised. |
| 10. Modifications | - Further to a protocol amendment, from the start of the intervention period children who failed to provide the completed parental ATS report on a draw day were able to take part based on their own report. |
| 11. How well (fidelity) | - All eight sessions were delivered as intended, but one had to be re-scheduled to a different day on one occasion due to other classroom commitments. |
